# Dual-Input and Multi-Channel Convolutional Neural Network Model for Vehicle Speed Prediction

**DOI:** 10.3390/s21227767

**Published:** 2021-11-22

**Authors:** Jiaming Xing, Liang Chu, Chong Guo, Shilin Pu, Zhuoran Hou

**Affiliations:** 1 State Key Laboratory of Automotive Dynamic Simulation and Control, Jilin University, Changchun 130022, China; xingjm19@mails.jlu.edu.cn (J.X.); chuliang@jlu.edu.cn (L.C.); guochong@jlu.edu.cn (C.G.); pusl20@mails.jlu.edu.cn (S.P.); 2 Research Institute, Jilin University, Yibin 644500, China

**Keywords:** speed prediction, vehicle signals, CNN, ECMS

## Abstract

With the development of technology, speed prediction has become an important part of intelligent vehicle control strategies. However, the time-varying and nonlinear nature of vehicle speed increases the complexity and difficulty of prediction. Therefore, a CNN-based neural network architecture with two channel input (DICNN) is proposed in this paper. With two inputs and four channels, DICNN can predict the speed changes in the next 5 s by extracting the temporal information of 10 vehicle signals and the driver’s intention. The prediction performances of DICNN are firstly examined. The best RMSE, MAE, ME and R^2^ are obtained compared with a Markov chain combined with Monte Carlo (MCMC) simulation, a support vector machine (SVM) and a single input CNN (SICNN). Secondly, equivalent fuel consumption minimization strategies (ECMS) combining different vehicle speed prediction methods are constructed. After verification by simulation, the equivalent fuel consumption of the simulation increases by only 4.89% compared with dynamic-programming-based energy management strategy and decreased by 5.40% compared with the speed prediction method with low accuracy.

## 1. Introduction

Vehicle speed prediction refers to the estimation of future vehicle speed with available information. By utilizing the predicted vehicle speed, the vehicle controller can adopt appropriate control strategies and the driver can adjust the manipulation in advance [1,2]. At present, vehicle speed prediction has become one of the research focuses in the field of intelligent vehicles as well as intelligent transportation, and is widely used in vehicle path planning, collision warning, advanced driver assistance systems, etc. [3,4,5]. However, vehicle speed prediction is affected by various factors such as human, vehicle, and road and traffic conditions [6]. The characteristics of time-varying and nonlinearity increase the complexity and difficulty of vehicle speed prediction. Improving the accuracy of vehicle speed prediction has important theoretical value and wide application prospects.

The current research methods for vehicle speed prediction can be generally divided into a model-based method and a data-based method. Model-based methods include the microscopic model, macroscopic model, etc. The microscopic model focuses more on the speed variation of each vehicle. Differential equations are established to simulate vehicle traveling. The speed can be updated by continuously solving the differential equations. Ghandour et al. proposed a new method for predicting the future vehicle motion state, including longitudinal speed, which was applied to predict potential danger and achieved satisfactory results [7]. Ma, Z et al. proposed a novel method to identify the normal load of each tire in a heavy truck in order to estimate the longitudinal velocity. The simulation showed that the predicted velocity could follow the trend of the real velocity and no significant lag was found [8]. The purpose of the microscopic model is not to predict vehicle speed with high accuracy, but to analyze vehicle dynamics in conjunction with speed prediction trends. The macroscopic model takes the vehicle group as the object of study and focuses more on the statistical characteristics of traffic flow. Magliolo et al. proposed a multiclass freeway traffic model that was applied to estimate the average speed of traffic for different classes of vehicles. Good prediction results were obtained in a test on a section of the Italian freeway [9]. Bie et al. proposed a macroscopic traffic prediction model that took weather into account. It was verified that the accuracy of prediction was improved compared with the traditional model [10]. The macroscopic model usually focuses on considering the average speed of a road or area. Therefore, it is difficult to provide accurate information about the speed variation of individual vehicles.

The model-based speed prediction method has fast parameter learning speed and does not require large data sets. However, its weak fitting ability leads to difficulties in constructing accurate mathematical models for highly stochastic vehicle speed.

The data-based speed prediction method can improve the ability to fit functional relationships between variables by utilizing large amounts of data. It does not make excessive assumptions about the prediction model and is free to learn arbitrary forms of mapping relationships from the training data. The most commonly applied data-based methods include stochastic prediction [11], shallow learning [12] and deep learning [13]. The most commonly applied method in stochastic prediction is the Markov chain constructed on the basis of the Markov process. The Markov process is a class of stochastic processes with a non-aftereffect property. The term “non-aftereffect” means that changes in the system state after the current phase are related only to the current phase and not to all previous phases. Shin et al. proposed a speed prediction algorithm based on fuzzy Markov chains to solve the problem of increasing computational power, which reduces the computational time by 85.5% while maintaining 99.1% prediction accuracy [14]. Zhou et al. proposed a vehicle speed prediction method based on a self-learning multi-step Markov model. The simulation results showed that the average prediction error was reduced by 25.70% compared with the traditional Markov predictor [15]. The Markov model assumes that the probability of a vehicle state change is kept fixed. If the driving condition differs significantly from the historical data, the prediction effectiveness of Markov chain decreases markedly.

The main purpose of shallow learning is to construct a data-driven mathematical model that finds the mapping between the input set and the output set. Common shallow learning methods are neural networks [1,6,16], support vector machines [4,17], extreme learning machines [18,19], etc. Zhang et al. developed a time-based neural network model and a distance-based neural network model for vehicle speed prediction from two perspectives. The results showed that the predicted speed profiles of the two models matched the variation of the actual speed profiles [6]. Li et al. combined the niche immune genetic algorithm-support vector machine (NIGA-SVM) prediction algorithm and genetic algorithm-support vector machine (GA-SVM) prediction algorithm, which can greatly improve the accuracy and timeliness of vehicle speed prediction [4]. These models have had great success in both theoretical analysis and application. The models of shallow learning are limited by finite samples and computational units. When it comes to representing complex functions, they are not competent.

The purpose of deep learning is the same as shallow learning. The difference is that deep learning enables complex function approximation by building a deep nonlinear network structure. Commonly applied deep learning methods include the convolutional neural network (CNN) and the long short-term memory network (LSTM). Song et al. constructed a CNN-based traffic prediction model utilizing time data and speed data collected on the main roads in Seoul, Korea, and verified that the prediction performance of the model was higher than that of the other two multi-layer perceptron models [20]. Han et al. combined a one-dimensional convolutional neural network with a bidirectional long short-term memory network to predict vehicle speed utilizing the information provided by V2V and V2I communication, and the model proposed significantly improved the prediction accuracy of short-term speed prediction compared with traditional shallow learning [21]. Both CNN and LSTM have achieved excellent prediction results in multiple fields. In terms of extracting data features, CNN is a master of extracting regional features and LSTM does well in extracting temporal features.

The data-based speed prediction method can theoretically fit a highly nonlinear mapping. However, low-value data can cause the model to learn wrong mappings. Therefore, it is important to collect diverse experimental data and explore the deep value of the data.

In this context, a dual-input CNN model (DICNN) is proposed in this paper. DICNN can extract features from vehicle signal sequences and driver intent to predict future speed changes. Firstly, road experiments are designed to collect rich vehicle data for a parallel plug-in hybrid electrical vehicle (PHEV). Secondly, the performance of DICNN is discussed compared with the other three prediction models. Finally, the prediction models are combined with the energy management strategy to further verify the relationship between the accuracy of prediction and the effect of energy saving.

The main innovations and contributions of this work are concluded as follows:

(1) Deep learning models are used to build the speed prediction model. The deep architecture has more tunable parameters, which can lead to better prediction results.
(2) The training set is constructed based on rich vehicle data obtained from road experiments. The various vehicle signal sequences provide a more detailed picture of the current operating status of the vehicle.
(3) The second type of input for the model characterizes the pedal signal. As a direct representation of driver intent in vehicle signals, the second type of input enables the prediction model to more sensitively capture trends in vehicle speed changes.

The paper is organized as follows. Section 2 describes the road experiments. Section 3 introduces the structure of CNN and DICNN. Section 4 presents the energy management strategy applied in this paper for vehicle simulation validation and introduces the method combined with vehicle speed prediction. Section 5 presents the model performance and simulation results. Finally, Section 6 concludes the paper.

## 2. Road Experiments

**Figure 1:**
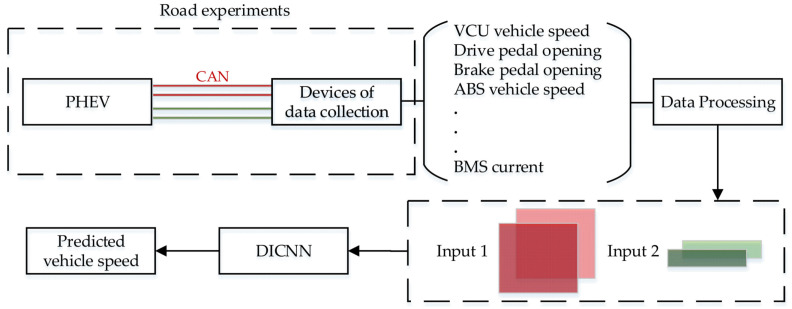
Flow chart of vehicle speed prediction.

Road experiments are designed to collect vehicle traveling data. The CAN bus signals of the vehicle are obtained from the devices of data collection. From all signals, 10 vehicle signals are selected and converted into a format that meets the input requirements of DICNN. The predicted vehicle speed is output based on the model. The whole flow is shown in Figure 1.

### 2.1. Vehicle Model

**Figure 2:**
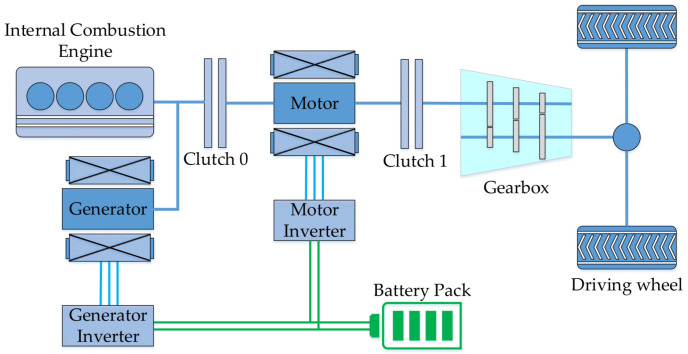
The schematic of the PHEV configuration.

The research object of this paper is a parallel PHEV. According to the placement of the electrification component, the vehicle belongs to the P2 type, i.e., the motor is placed behind the clutch 0 and before the transmission [22]. The vehicle configuration is shown by Figure 2. When the clutch 0 is disengaged, the motor drives the vehicle alone, avoiding the engine operating in the inefficiency area and allowing for brake energy recovery. When the clutch 0 is engaged, the engine and motor work together [23]. The switching conditions between modes are mainly determined by the overall vehicle demand power and the state of charge (SOC) [24]. Vehicle and component parameters are shown by Table 1.

### 2.2. Experiment Method

**Figure 3:**
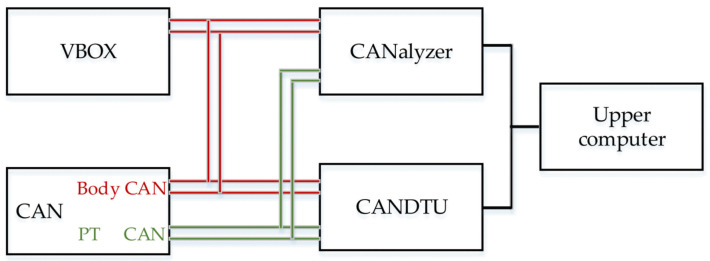
The schematic of data collection.

We apply the on-board recorder to collect vehicle signals. In order to evaluate the data quality of the on-board recorder and provide an accurate basis for processing, VBOX is applied to evaluate and calibrate the key information such as vehicle speed and acceleration. The hardware scheme is shown in Figure 3. The VBOX with a GPS antenna and an acceleration sensor sends the collected key signals such as latitude, longitude, speed and acceleration to the CANalyzer and the CANDTU as CAN signals. CANalyzer is a versatile analysis tool for network and distributed system development [25]. It enables easy observation and analysis of data transmission [26]. CANDTU records packet information in the CAN bus for an extended period of time, for subsequent data processing. Body CAN and powertrain CAN (PT CAN) are displayed and recorded in real time by the upper computer.

In order to reduce the stress on data storage, computation and signal filtering, the signal list was improved as shown in Table 2. The VBOX-related signals were added. The signals related to the energy consumption calculation of each assembly and component were retained. ABS vehicle speed, VCU vehicle speed, VBOX vehicle speed and VBOX acceleration were adopted for redundant calculations to improve speed and acceleration acquisition accuracy.

### 2.3. Data Processing

Due to the different signal frequencies, noise, abnormal data, etc., the collected data need to be processed first. The basic steps include data synchronization, filtering, etc. Due to the different sampling frequencies, the different signals are not sampled at strictly the same time. The time stamp of the VCU vehicle speed is used as the reference to normalize the sampled time of other signals.

The vehicle system is a continuous system. The derivatives of vehicle signals such as vehicle speed, for example, are still physically significant and should not have abrupt changes. The Hermite interpolation polynomial ensures that the polynomial has equal function values and derivative values at the interpolation nodes. For long time sequences, the degree of the polynomial obtained by Hermite interpolation is high, and there is also the Runge phenomenon. Therefore, the segmented cubic Hermite interpolation is adopted in this paper, and its formula is shown as follows.

(1) $$\begin{array}{l} I_{h}(x)={\left(\frac{x-x_{k+1}}{x_{k}-x_{k+1}}\right)}^{2}\left(1+2\frac{x-x_{k}}{x_{k+1}-x_{k}}\right)f_{k}+{\left(\frac{x-x_{k}}{x_{k+1}-x_{k}}\right)}^{2}\left(1+2\frac{x-x_{k-1}}{x_{k}-x_{k-1}}\right)+\\ \;\;\;\;\;\;\;\;\;\;\;\;\;\;\;{\left(\frac{x-x_{k+1}}{x_{k}-x_{k+1}}\right)}^{2}\left(x-x_{k}\right)f_{k}^{\prime }+{\left(\frac{x-x_{k}}{x_{k+1}-x_{k}}\right)}^{2}\left(x-x_{k+1}\right)f_{k+1}^{\prime } \end{array}$$

where $I_{h}(x)$ is the cubic polynomial on the interval of $\left[x_{k},x_{k+1}\right]$; $f_{k}$ is the function value at the node k; and $f_{k}^{\prime }$ is the derivative value of the node k.

In the process of data processing, some data are beyond a reasonable range, such as the acceleration calculated from the vehicle speed. They generally behave as non-operational achievable high frequency jitter. Therefore, a recursive mean filter is adopted for the data process. Its filtering rule is that low frequency signals can pass normally, while high frequency signals exceeding the set frequency threshold are blocked and attenuated. The pseudo code of the recursive mean filter is shown in the Algorithm 1.
**Algorithm 1.** Recursive mean filter
**Data**: origin signal vector S, vector length L, window length N
**Result**: filtered signal vector S′1 **for** i ← 1**to**L **do**2  **if** i+N−1>L **then** break3  **Else then**
S′[i] ← mean(S[i,...,N+i−1])4  **end**5 **end**


## 3. Speed Prediction Model

### 3.1. CNN

In 1962, Hubel and Wiesel discovered that the unique network structure of neurons used for local sensitivity and orientation selection in the cat cerebral cortex could effectively reduce the complexity of feedback neural networks, and then proposed CNN. In recent times, CNN has become a key focus for research in many scientific fields. Its layout is closer to that of an actual biological neural network, and weight sharing reduces the complexity of the network. In particular, images with multidimensional inputs can be fed directly to the network, which avoids the complex data reconstruction during feature extraction. Its structure consists of an input layer, an intermediate layer and an output layer, where the intermediate layer includes a convolutional layer, pooling layer, activation function and batch normalization layer.

#### 3.1.1. Convolution Layer

The role of the convolution layer is compression and purification in order to enhance input signals and reduce noise. The equation for the convolution layer is described by

(2) $$z^{\left(l\right)}=w^{\left(l\right)}⊗x^{\left(l-1\right)}+b^{\left(l\right)}$$

where l is the number of the layer; w is a convolution kernel; x is the activity value; b is a learnable bias; and z is the output of convolution layer. The neurons between the convolutional layers are locally connected and share the weights [27]. The characteristics greatly speed up the training and decrease the number of weights and biases. Local connectivity means that the neurons in the convolution layer are connected to the neurons in the next layer only in a local area, which form a locally connected network.

#### 3.1.2. Batch Normalization Layer

To speed up the training of convolutional neural networks and reduce the sensitivity to network initialization, a batch normalization layer is employed, which normalizes each input channel in a small batch [28]. The strategy of batch normalization is to first compute the sample mean and sample variance, and then perform normalization, translation and scaling on the sample data. The equations are described by Equations (3)–(6).

(3) $$\mu_{B}=\frac{1}{m}\sum_{i=1}^{m}x_{i}$$

(4) $$\sigma_{B}^{2}=\frac{1}{m}\sum_{i=1}^{m}{\left(x_{i}-\mu_{B}\right)}^{2}$$

(5) $${\hat{x}}_{i}=\frac{x_{i}-\mu_{B}}{\sqrt{\sigma_{B}^{2}+\varepsilon }}$$

(6) $$BN_{\gamma ,\beta }(x_{i})=\gamma {\hat{x}}_{i}+\beta$$

where x is the sample; m is the number of elements in the sample; $\mu_{B}$ is the sample mean; $\sigma_{B}^{2}$ is the sample variance; ε is an infinitesimal positive number; and γ and β are training parameters.

#### 3.1.3. Activation Layer

The activation layer is responsible for activating the features extracted from the convolution layer. Since the convolution operation is a linear transformation, it is necessary to introduce an activation layer belonging to a nonlinear function for nonlinear mapping. The ReLU and the Softsign functions are applied. The ReLU function performs a threshold operation to set any input less than zero to zero [29]. The Softsign function has the flatr curve and slow decreasing derivatives for more efficient learning [30]. Their calculation equations are shown by Equations (7) and (8), respectively.

(7) $$ReLU(x)=\left\{\begin{array}{ll} x & x\geq 0\\ 0 & x<0 \end{array}\right.$$

(8) $$Softsign(x)=\frac{x}{1+\left|x\right|}$$

#### 3.1.4. Pooling Layer

The pooling layer, also known as the subsampling layer, serves to perform feature selection and reduces the number of features. The pooling layer itself does not require any learning, but it reduces the number of parameters to be learned in the subsequent layers and also helps to reduce overfitting. The pooling function adopted in this paper is average pooling. For a region, the average pooling calculates the mean of all neurons in this region as the representation of this region, as shown in Equation (9).

(9) $$y_{m,n}^{d}=\frac{1}{\left|R_{m,n}^{d}\right|}\sum_{i\in R_{m,n}^{d}}x_{i}$$

where $R_{m,n}^{d}$ is the selected region; $x_{i}$ is the value in $R_{m,n}^{d}$; and $y_{m,n}^{d}$ is the representation of $R_{m,n}^{d}$.

#### 3.1.5. Fully Connected Layer

Each node in the fully connected layer is connected to all nodes in the previous layer, which combines the features extracted from the previous layer. For the vehicle speed prediction model belonging to the regression model, the output size of the fully connected layer is set to 1 which is the future speed of the vehicle. In this paper, five models with different parameters are trained to predict the future speed from 1 to 5 s in turn.

### 3.2. The Architecture of DICNN

**Figure 4:**
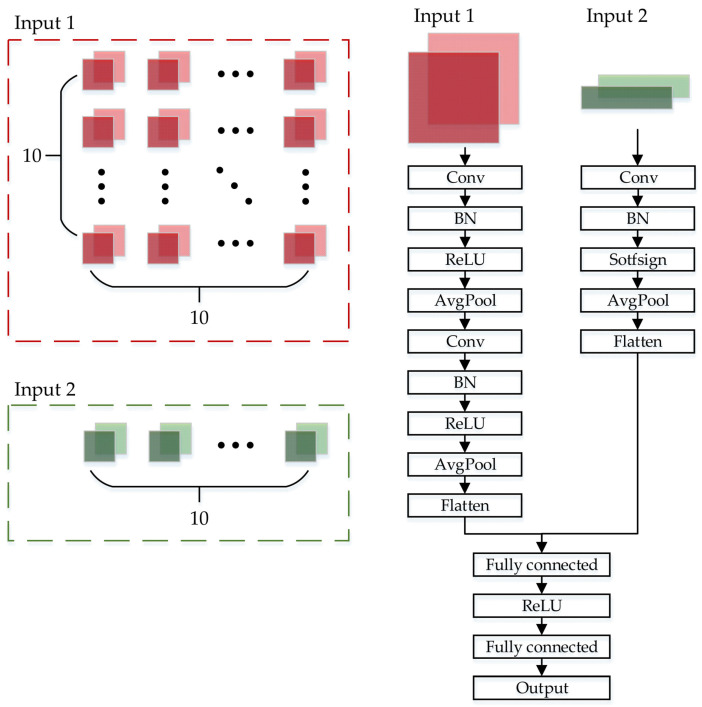
The architecture of DICNN.

The architecture of DICNN is shown in Figure 4. DICNN has two types of input and a total of four channels.

The first type of input consists of two channels. Each channel contains the 10*10 fake images. The first type of input reflects the characteristics of multiple vehicle signals at short-term adjacent moments. Its first channel adopts a fake image composed of 10 s timing vectors of 10 vehicle signals. The signals selected in this paper are VCU vehicle speed, VBOX vehicle speed, longitudinal acceleration, motor speed, motor torque, DC bus voltage, DC bus current, state of charge, BMS voltage and BMS current. The second channel adopts the horizontal gradient map of the fake image. The gradient map can describe the local intensity gradient or image edge well, and is a common method in image feature extraction. It is obtained by performing gradient operation on the image. For the fake image, the gradient in the horizontal direction has practical physical meaning. Extracting the characteristics of the signal gradient changes helps to provide a reference for future vehicle speed changes. Therefore, the horizontal gradient map of the first channel is used as the input of the second channel.

The second type of input also consists of two channels, each containing a 1 × 10 vector to characterize the driver’s driving intentions. The driver’s intention is directly represented in the vehicle signal by the drive pedal opening and brake pedal opening. The drive pedal on is scaled from 0 to 1 and the brake pedal on is scaled from −1 to 0. The drive pedal opening and brake pedal opening are combined into one vehicle signal named pedal opening. The temporal pedal openings and their temporal accumulation values are used as the two channels of the second type of input to extract driving intentions and predict speed changes. The format of the input to DICNN is shown by

(10) $$\left\{\begin{array}{c} P_{1}^{i,j,1}{=S}_{i}^{j}\;\;\;\;\\ P_{1}^{i,j,2}=\frac{S_{i}^{j}-S_{i-1}^{j}}{\Delta t}\\ P_{2}^{i,1,1}=\gamma_{i}\\ P_{2}^{i,1,2}=\sum_{m=1}^{i}\gamma_{m} \end{array}\right.\;\;\;\;\;\;\;\;(i\in I_{s},j\in [1,2,...,10])$$

where $P_{1}$ and $P_{2}$ are the first type and the second type of the input of DICNN, respectively; i is the number of sampling points; $S^{j}$ represents the type of signal, from 1 to 10, in order of VCU vehicle speed, VBOX vehicle speed, longitudinal acceleration, motor speed, motor torque, DC bus voltage, DC bus current, state of charge, BMS voltage and BMS current, respectively; Δt is the time interval between two adjacent sampling points; and γ is the pedal opening. Data set allocations are established: 70% of the data sets are allocated for training, 15% for validation, and the remaining 15% for testing.

## 4. Energy Management Strategy

PHEV improves the fuel economy by applying both fuel and electricity. In order to meet the vehicle’s power requirements while optimizing the vehicle economy, the control strategy needs to adjust the power distribution between the engine and electrical energy at any given moment according to the real-time road conditions. The development of a reasonable energy management strategy is the basis for achieving better economy for PHEV. This section analyzes the calculation principle of the equivalent fuel consumption minimization strategy (ECMS) and combines it with vehicle speed prediction for optimization.

### 4.1. Fundamentals of ECMS

ECMS is derived from Pontryagin’s minimum principle (PMP) [31]. Combined with empirical engineering knowledge, it has become a semi-analytic approach to optimal energy management [32]. This principle allows to determine the optimal control decision based on the boundary conditions of the chosen mathematical model in the case of a continuously running system. Further, the optimal trajectory of the state variables of the system and the optimal covariates are solved. When PMP is applied to the energy management of PHEV, the Hamiltonian equation is expressed by

(11) $$H(t)=P_{fuel}(t)+s\cdot P_{elec}(t)$$

where H is the power cost; s is the co-state variable which is equivalent to a weight; $P_{elec}$ is the electrochemical power of the battery; and $P_{fuel}$ is the fuel consumption power, which has an energy conversion relationship with the engine power, as shown in the Equation (12).

(12) $$P_{fuel}(t)=\frac{P_{e}(t)}{\eta_{e}(t)}$$

where $P_{e}$ is the engine power; and $\eta_{e}$ is the engine efficiency. Similarly, there is an energy conversion between the electrochemical power and the electrical power of the battery. When the battery is discharged, the process of converting the chemical energy into electrical energy generates energy loss as shown in Equation (13).

(13) $$P_{elec}(t)=\frac{P_{b}(t)}{\eta_{b}(t)}$$

where $P_{b}$ is the battery power; and $\eta_{b}$ is the battery efficiency. Substituting Equation (12) and Equation (13) into Equation (11) yields Equation (14).

(14) $$H(t)=\frac{P_{e}(t)}{\eta_{e}(t)}+s\cdot \frac{P_{b}(t)}{\eta_{b}(t)}$$

The control objective is to minimize the equivalent fuel consumption at each moment. Therefore, the dependent variable of the Hamiltonian function should be transformed into the instantaneous equivalent fuel consumption. By dividing both sides of Equation (14) by the calorific value of the fuel at the same time, Equation (15) is obtained.

(15) $$\frac{dm_{eq}}{dt}=\frac{P_{e}(t)}{\eta_{e}(t)\cdot Q_{fh}}+s\cdot \frac{P_{b}(t)}{\eta_{b}(t)\cdot Q_{fh}}=\frac{dm_{fuel}}{dt}+s\cdot \frac{dm_{elec}}{dt}$$

where $\frac{dm_{eq}}{dt}$ is the instantaneous total equivalent fuel consumption rate; $\frac{dm_{fuel}}{dt}$ and $s\cdot \frac{dm_{elec}}{dt}$ are the fuel consumption rate of the engine and the equivalent fuel consumption rate of the battery, respectively. The different types of energy are unified into the same form for calculation. The ratio of engine and battery power that minimizes the instantaneous total equivalent fuel consumption rate is chosen as the current optimal solution.

### 4.2. ECMS with Speed Prediction

The optimization objective of the conventional ECMS is to minimize the equivalent fuel consumption at the current moment. The optimization objective of the ECMS with speed prediction is to minimize the sum of the fuel consumption of the engine and the equivalent fuel consumption of the motor at each moment in the predicted time domain. At each sampling moment, an open-loop optimization problem in a finite time domain is solved based on the obtained vehicle information. The first element of the obtained control sequence is applied to the controlled object. At the next sampling moment, the above process is repeated to complete the control of the energy management strategy.

In this paper, a dynamic programming (DP) algorithm is adopted to solve the optimization problem. The basic idea of the dynamic programming algorithm is to transform a multi-stage problem into multiple sub-problems of the same type, and gradually recursively search for optimization by solving the optimal solution of the sub-problems [33]. The algorithm is based on the Behrman optimization principle. In any state of a multi-stage globally optimal control strategy, regardless of its past states and decisions, the remaining many decisions must constitute an optimal sub-strategy. In short, any part of the sub-strategies in the optimal strategy must be optimal.

In this paper, the optimization task is described in terms of travel time. The sampling interval is set to 1 s, i.e., $T_{s}=1$. The ratio of the total duration of the driving cycle to the sampling interval is taken as the phase of the optimization problem, i.e., $N=T/T_{s}$. The state variable, control variable and parameters are set as described in Equation (16).

(16) $$\begin{array}{l} x=SOC\\ u=\lambda \\ p=[v,a,gear]{\;}^{T} \end{array}$$

where x is the state variable; u is the control variable; p is the parameters; SOC is the state of charge of the battery; λ is the ratio of motor power to vehicle demand power; and v, a and gear are the speed, acceleration and gear number, respectively, of the vehicle. The state variable is updated as shown in Equation (17).

(17) $$SOC(k+1)=SOC(k)-\frac{I_{b}(u,p)\cdot T_{s}}{3600C_{b}}$$

where k is the current stage; $I_{b}$ is the battery current; and $C_{b}$ is the battery capacity. The cost function is shown by Equation (14). At this point, a complete energy management strategy is constructed.

## 5. Validation

In this paper, the prediction effectiveness of DICNN is examined from both performance evaluation and vehicle simulation in turn. Three other speed prediction methods are selected for comparison and validation, which are Markov chain combined with Monte Carlo (MCMC), support vector machine (SVM) and single input CNN (SICNN). Both MCMC and SVM only use the vehicle speed with a history of 10 s as the model input. SICNN only usea the first type of input of DICNN as model input. The driving cycle adopted for validation is the worldwide harmonized light vehicles test cycle (WLTC).

### 5.1. Preformance Evaluation

**Figure 5:**
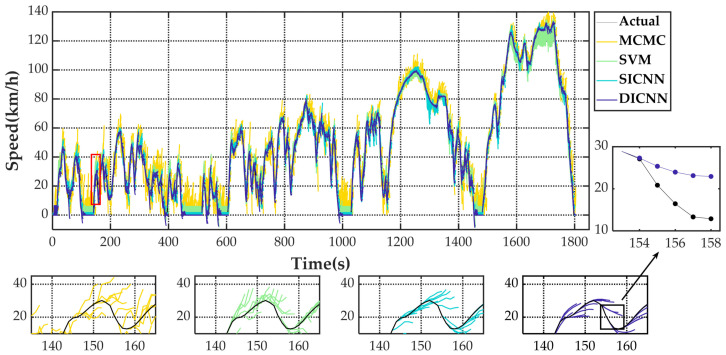
The speed prediction results of four methods for WLTC. The dots (lines) with color are predicted values and the black dots (lines) are actual values.

**Figure 6:**
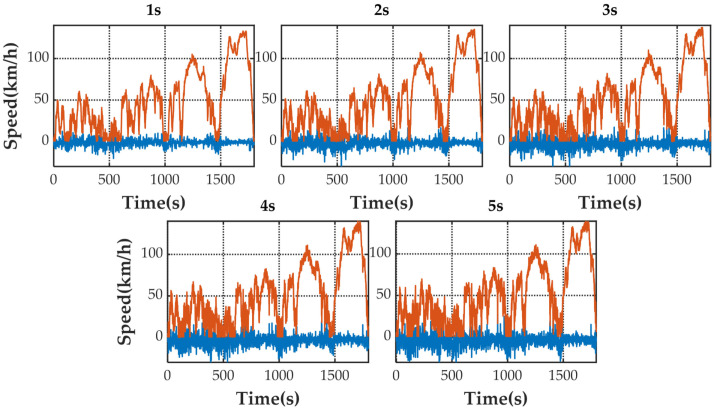
The prediction error (blue) and prediction speed (orange) of MCMC.

**Figure 7:**
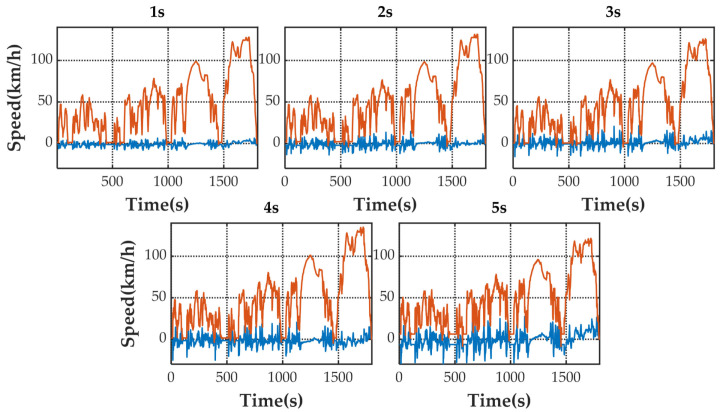
The prediction error (blue) and prediction speed (orange) of SVM.

**Figure 8:**
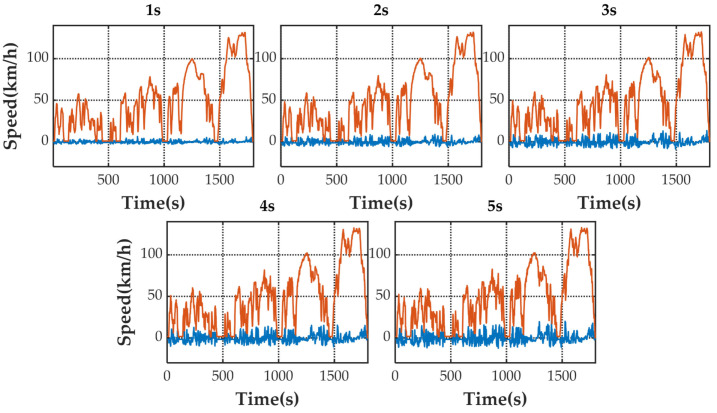
The prediction error (blue) and prediction speed (orange) of SICNN.

**Figure 9:**
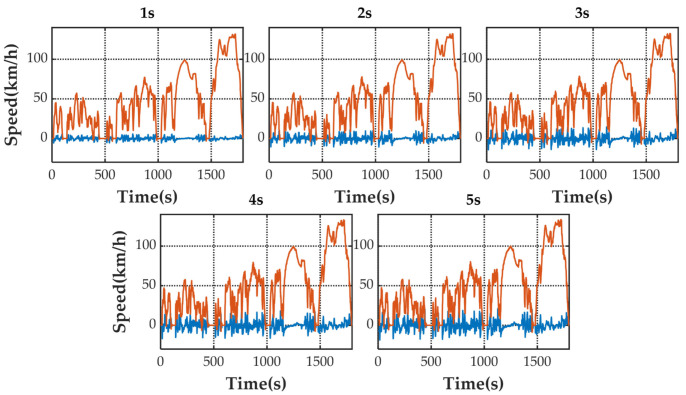
The prediction error (blue) and prediction speed (orange) of DICNN.

**Figure 10:**
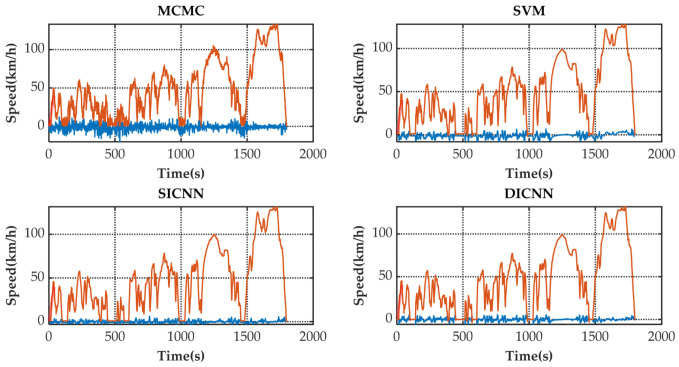
The prediction error (blue) and prediction speed (orange) for the next 1 s.

**Figure 11:**
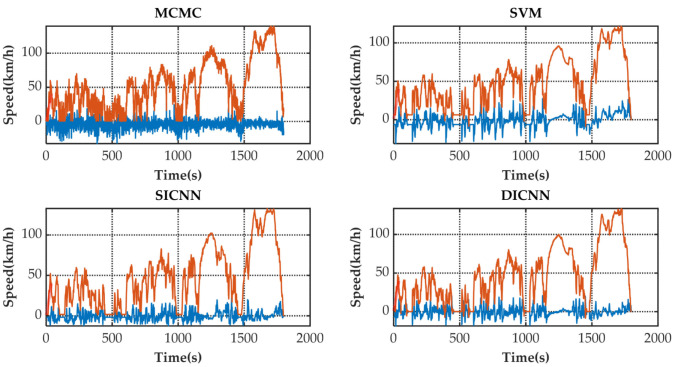
The prediction error (blue) and prediction speed (orange) for the next 5 s.

**Figure 12:**
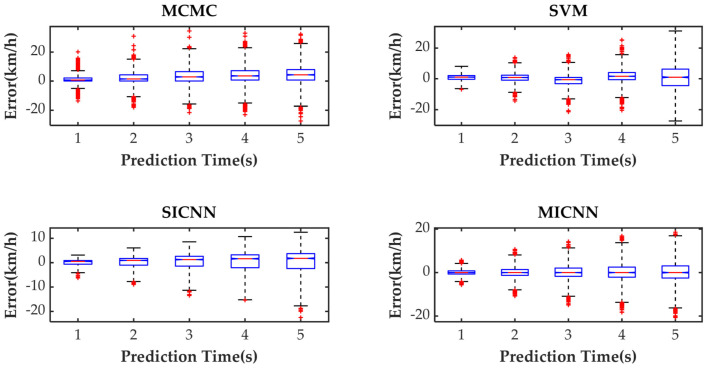
The box plots of the prediction errors.

**Figure 13:**
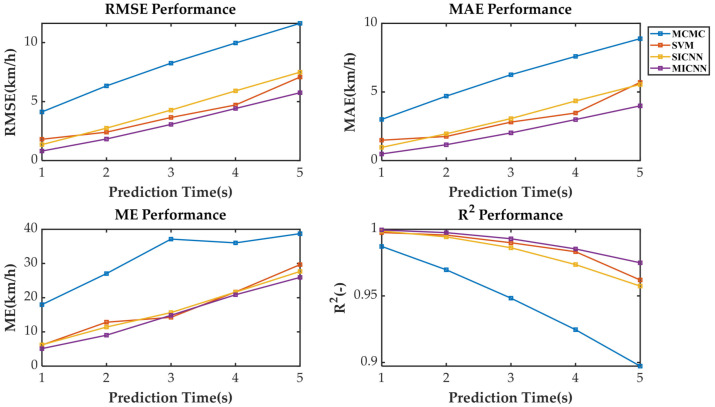
The performances of the four methods.

Figure 5 shows the speed prediction results of four methods for WLTC. Figure 6, Figure 7, Figure 8 and Figure 9 show the prediction error of MCMC, SVM, SICNN and DICNN, respectively. Figure 10 and Figure 11 show the prediction error for the next 1 s and the next 5 s. Figure 12 shows the box plot of prediction error. Figure 13, Table 3, Table 4, Table 5 and Table 6 show the performances of four methods.

The black line in Figure 5 is the vehicle speed of WLTC. Each colored line is the prediction result of 5 s after the moment corresponding to the beginning of the line. Figure 5 shows that yellow lines have the widest distribution, which represents that MCMC has the largest prediction error. The prediction results of SVM are better than MCMC, but there are larger prediction errors at high speed. The coverage of SICNN and DICNN is similar. However, more deviations in the lines of SICNN can be observed in some moments when the acceleration and deceleration processes switch. As can be seen from the partial enlargement of Figure 5, MCMC has a strong randomness in the predicted speed change due to the randomness of the process of moving from one state to another in the state space. The detail view of SVM has more regular prediction results compared with MCMC because SVM belongs to supervised learning. SICNN can grasp the acceleration variation of the vehicle more precisely after utilizing multiple vehicle signals. In combination with pedal opening, DICNN is more sensitive to speed changes and obtains smoother prediction curves than SICNN. The detail view of DICNN shows that the future state of the vehicle can be predicted before the vehicle state changes, which indicates that DICNN can find a more exact mapping relationship between input and output.

Figure 6, Figure 7, Figure 8 and Figure 9 show the prediction error and predicted vehicle speed of MCMC, SVM, SICNN and DICNN in turn. Figure 10 and Figure 11 compare all methods for the same prediction horizon. Firstly, the prediction errors of different time horizons are compared. It can be seen that the coverage of the blue curve increases for all methods as the prediction horizon increases. This represents a gradual increase in prediction error and a significant decrease in prediction accuracy. Comparing the high-speed prediction with the low-speed prediction, the former predicts better results than the latter for all methods and all horizons. The results arise from the fact that low-speed conditions are mostly caused by environmental constraints which lead to highly complex calculations and difficulties for prediction. Comparing the different methods, the prediction of MCMC is the worst among all methods, followed by SVM. There is no significant difference between SICNN and DICNN observed only from the figures.

Box plots are used to reflect the characteristics of the data distribution. Figure 12 shows the box plots for different prediction methods and horizons. On each box, the central mark indicates the median, and the bottom and top edges of the box indicate the 25th and 75th percentiles, respectively. The whiskers extend to the most extreme data points not considered outliers, and the outliers are plotted individually using the ‘+’ symbol. As the prediction horizon increases, both the bottom and top edges of the box gradually move away from 0 km/h, and the outliers gradually decrease with greater coverage between the bottom and top edges. The prediction errors of SICNN have more negative values, which means that SICNN is inclined to predict a high probability of braking or decelerating actions of the vehicle in the future.

Table 3, Table 4, Table 5 and Table 6 show the performances of the different methods and horizons. Figure 13 shows the results graphically. Root mean square error (RMSE), mean absolute error (MAE), maximum absolute error (ME) and R-square (R^2^) are popular evaluation indicators for the prediction results. When the predicted speed is close to the actual speed, RMSE, MAE, ME and R^2^ are close to zero. Conversely, R^2^ close to 1 indicates an excellent match. It can be precisely analyzed that DICNN outperforms the other three speed prediction methods in terms of MAE, RMSE and R^2^ at all horizons except the third second ME.

### 5.2. Simulation

**Figure 14:**
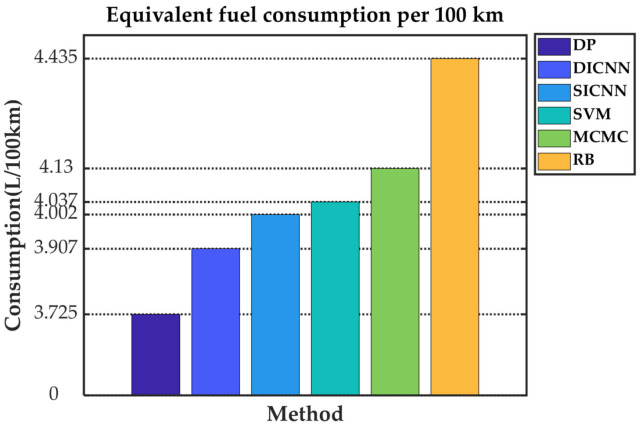
Equivalent fuel consumption per 100 km.

**Figure 15:**
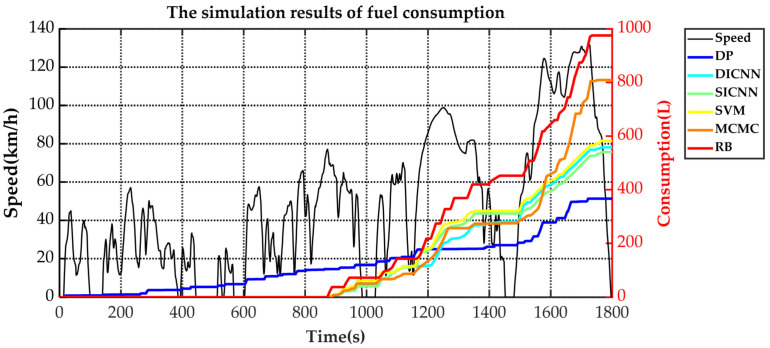
The changes of fuel consumption under various strategies.

**Figure 16:**
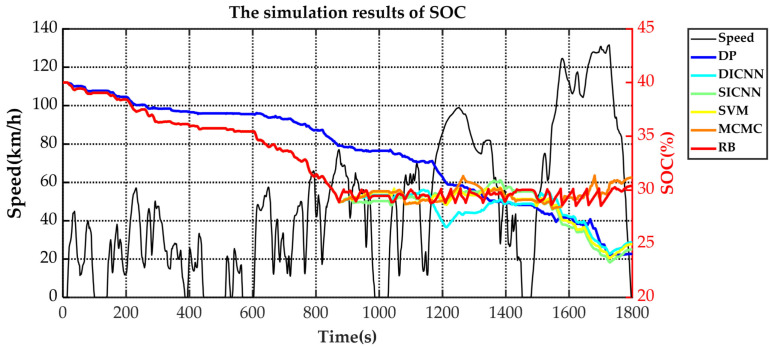
The changes of SOC under various strategies.

**Figure 17:**
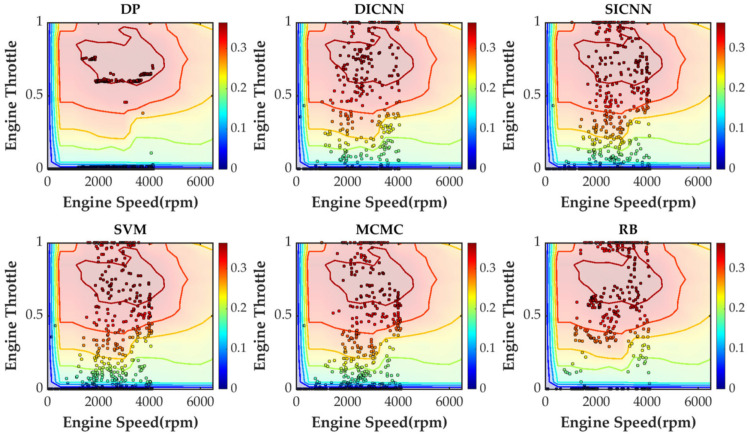
The distribution area of engine operating points under various strategies.

**Figure 18:**
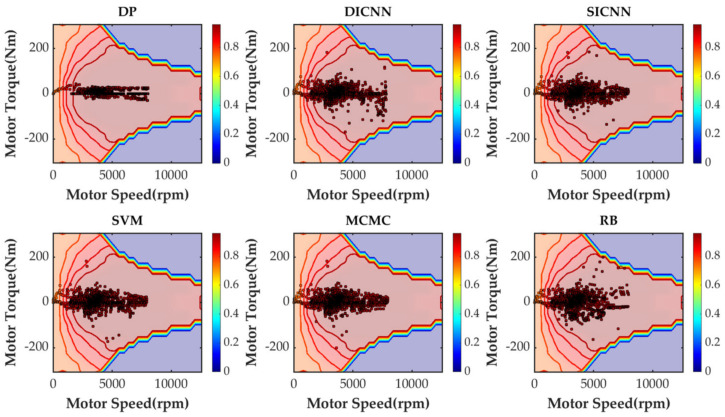
The distribution area of motor operating points under various strategies.

**Figure 19:**
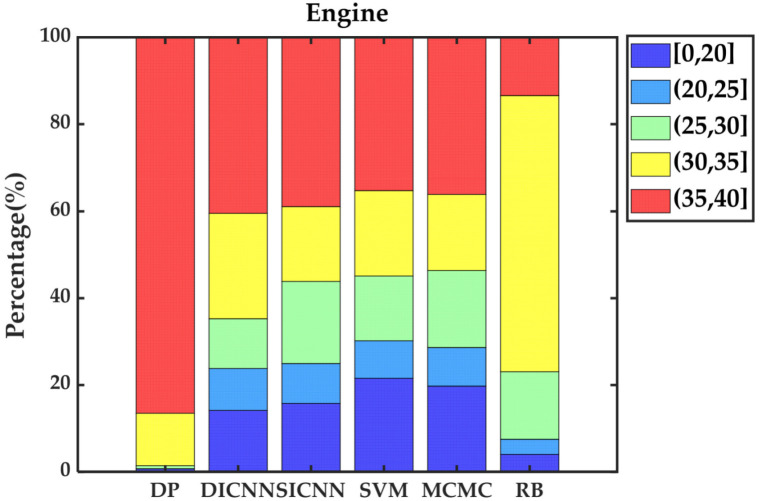
The number of engine operating points in each efficiency range.

**Figure 20:**
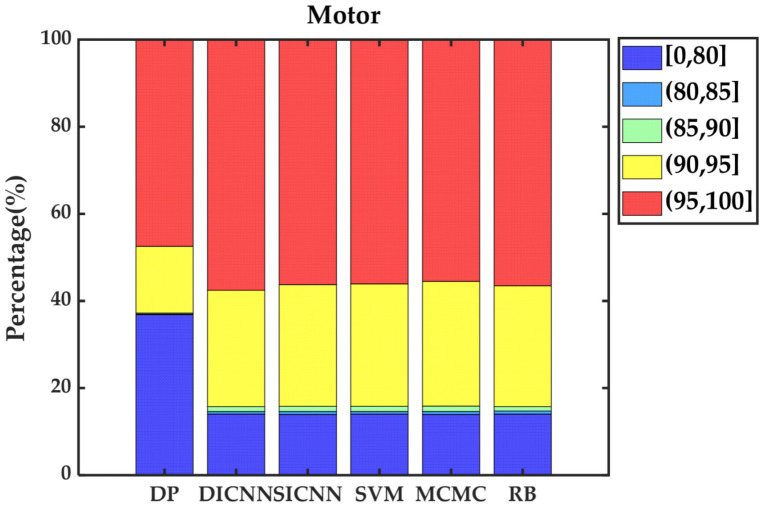
The number of motor operating points in each efficiency range.

Simulation validation is performed under the Matlab/Simulink platform. This paper establishes a rule-based energy management strategy (RB), a DP-based energy management strategy and ECMS-based energy management strategies adopting the vehicle speed prediction methods of MCMC, SVM, SICNN and DICNN. Figure 14 and Table 7 show the equivalent fuel consumption. Figure 15 shows the changes of fuel consumption. Figure 16 shows the changes of SOC. Figure 17 shows the distribution area of the engine operating points. Figure 18 shows the distribution area of motor operating points. Figure 19 shows the number of engine operating points in each efficiency range. Figure 20 shows the number of motor operating points in each efficiency range.

As can be seen in Figure 14 and Table 7, as a criterion of optimization effectiveness, DP has the best economy with an equivalent fuel consumption of only 3.725 L/100 km. DICNN-based ECMS, which has the best speed prediction capability, achieved the best ECMS-based optimization results. The equivalent fuel consumption of the DICNN-based ECMS is 3.907 L/100 km, which is 4.89% higher than DP. As the accuracy of speed prediction decreases, the number of control decisions that do not fit future conditions increases, resulting in an increase in equivalent fuel consumption. Since the rule-based energy management strategy does not have the ability to dynamically adjust the control strategy, the worst economy with an equivalent fuel consumption of 4.435 L/100 km is obtained.

The changes of fuel consumption in Figure 15 show that the DP starts the engine frequently to keep the fuel consumption slowly rising. DICNN-based ECMS, SICNN-based ECMS and SVM-based ECMS approximately maintain the same operating period and fuel consumption. The rule-based energy management strategy and MCMC-based ECMS adopt a high engine power and consume more fuel than other methods. Combined with Figure 16, results show that DP often starts the engine to assist the motor in sharing the vehicle demand power. Therefore, its SOC changes are also relatively flat and nearly linearly decreasing. Other methods use the charge depleting mode in the first half of the simulation and the charge sustaining mode in the second half because there is no global information about the driving cycle. DICNN-based ECMS, SICNN-based ECMS and SVM-based ECMS have a gentle curve and keep the motor running to take the main demand power at high speed conditions. The rule-based energy management strategy and MCMC-based ECMS has obvious upper and lower limits of the SOC fluctuation range. Therefore, at high speed conditions, fuel consumption increases sharply to ensure that SOC does not drop.

In order to further analyze the working condition of components, distributions of engine operating points and motor operating points are analyzed. From Figure 17 and Figure 18, points represent the operating points of components and colors represent the efficiency of components. DP provides the theoretically optimal control strategy and does not take into account the start/stop process of the engine. Most of engine operating points of DP are distributed in the high efficiency area of the engine. The rest of the methods contain the engine starting process and the pedal-pressing process, so the operating points are distributed in both high efficiency and low efficiency areas. ECMS, combining different speed prediction models, have similar distributions of operating points. The operating points of the rule-based energy management strategy are less distributed in both the high efficiency and low efficiency areas. With regard to the motor, DP has the most concentrated distribution of operating points and RB has the most dispersed distribution of operating points. The distributions of ECMS-based energy management strategies are all relatively similar.

Figure 19 and Figure 20 further show the number of operating points of the engine and motor distributed in different efficiency ranges. For the engine, the operating points of DP are almost entirely distributed in the area above 30% efficiency. In the ECMS methods, DICNN has the largest number of operating points in the high efficiency range and the lowest number of operating points in the low efficiency range, which is exactly the opposite of RB. DICNN-based ECMS, SICNN-based ECMS, SVM-based ECMS and MCMC-based ECMS are distributed in similar numbers in each range, where DICNN-based ECMS is closest to DP. For the motor, the distribution of operating points of DP is poor because DP focuses on adjusting the position of operating points of the engine in the high efficiency area. For the other methods, the distributions do not differ significantly. However, DICNN-based ECMS obtains the largest number of distributions in the 95–100% efficiency range by a narrow margin.

## 6. Conclusions

In conclusion, we propose a novel CNN-based neural network architecture named DICNN. With two inputs and four channels, DICNN can predict the speed change in the next 5 s by extracting the temporal information of 10 vehicle signals and the driver’s intention. It is compared with representative methods in stochastic prediction, machine learning and deep learning. The results show that DICNN outperforms other methods in terms of performance evaluation metrics including RMSE, MAE, ME and R^2^. We also construct ECMS energy management strategies with speed prediction for simulation and analysis. With the increase of prediction accuracy of vehicle speed, the difference between ECMS and DP can be reduced from 10.87% to 4.89%. More operating points of the power components are distributed in the high efficiency area to achieve lower equivalent fuel consumption.

In the future, we will explore the impact of the type and number of vehicle signals on the prediction effect. In addition, more deep-learning-based models will be combined to further extract features from the training data and improve the accuracy of prediction.

## Figures and Tables

**Table 1:** Vehicle and component parameters in the studied vehicle.

Component	Parameter	Value	Unit
Vehicle	Vehicle weight	1200	kg
	Tire radius	0.323	m
	Final drive ratio	4.021	/
Engine	Maximum torque	165	Nm
	Maximum power	105@6500	kW@rpm
Motor	Maximum torque	307	Nm
	Maximum power	126@12584	kW@rpm
Battery	Rated capacity	20.8	Ah
	Rated voltage	366	V
Gear	First	3.527	/
	Second	2.025	/
	Third	1.382	/
	Fourth	1.058	/
	Fifth	0.958	/

**Table 2:** The signal list.

Knot	Signal	Source	Frequency
VCU	VCU vehicle speed	PT CAN	100 Hz
	Drive pedal opening	PT CAN	100 Hz
	Brake pedal opening	PT CAN	100 Hz
ABS	ABS vehicle speed	Body CAN	100 Hz
VBOX	Latitude	Body CAN	100 Hz
	Longitude	Body CAN	100 Hz
	VBOX vehicle speed	Body CAN	100 Hz
	longitudinal acceleration	Body CAN	100 Hz
MCU	Motor speed	PT CAN	100 Hz
	Motor torque	PT CAN	100 Hz
	DC bus voltage	PT CAN	100 Hz
	DC bus current	PT CAN	100 Hz
ECU	Engine speed	PT CAN	100 Hz
	Engine torque	PT CAN	100 Hz
BMS	State of charge	PT CAN	10 Hz
	BMS voltage	PT CAN	10 Hz
	BMS current	PT CAN	10 Hz

**Table 3:** RMSE performance.

Method	Prediction Time (s)				
	1s	2s	3s	4s	5s
MCMC	4.12	6.33	8.25	9.96	11.63
SVM	1.81	2.41	3.66	4.71	7.08
SICNN	1.36	2.74	4.28	5.91	7.49
DICNN	0.81	1.83	3.07	4.41	5.75

**Table 4:** MAE performance.

Method	Prediction Time (s)				
	1s	2s	3s	4s	5s
MCMC	3.00	4.70	6.26	7.59	8.88
SVM	1.48	1.76	2.80	3.47	5.70
SICNN	0.97	1.96	3.06	4.35	5.55
DICNN	0.48	1.15	2.02	2.99	3.99

**Table 5:** ME performance.

Method	Prediction Time (s)				
	1s	2s	3s	4s	5s
MCMC	17.97	27.04	37.12	36.02	38.72
SVM	6.15	12.86	14.27	21.67	29.67
SICNN	6.22	11.43	15.66	21.63	27.68
DICNN	5.12	9.03	14.85	20.87	25.95

**Table 6:** R^2^ performance.

Method	Prediction Time (s)				
	1s	2s	3s	4s	5s
MCMC	0.9871	0.9696	0.9483	0.9246	0.8973
SVM	0.9975	0.9956	0.9899	0.9832	0.9619
SICNN	0.9986	0.9943	0.9861	0.9735	0.9574
DICNN	0.9995	0.9975	0.9929	0.9852	0.9749

**Table 7:** Simulation results.

Method	Fuel Consumption (mL)	Final SOC (%)	Equivalent Fuel Consumption (L/100 km)	Increased Equivalent Fuel Consumption Compared with DP (%)
DP	366.76	24.05	3.725	/
MICNN	558.19	25.13	3.907	4.89
SICNN	540.21	24.47	4.002	7.44
SVM	582.13	24.96	4.037	8.38
MCMC	809.30	31.16	4.130	10.87
RB	975.54	30.38	4.435	19.06
